# Stem-like T cells and niches: Implications in human health and disease

**DOI:** 10.3389/fimmu.2022.907172

**Published:** 2022-08-17

**Authors:** Linglu Yi, Li Yang

**Affiliations:** State Key Laboratory of Biotherapy and Cancer Center, West China Hospital, West China Medical School, Sichuan University and Collaborative Innovation Center of Biotherapy, Chengdu, China

**Keywords:** stem-like T cells, niches, adoptive cell therapies, autoimmune disorders, immune checkpoint blockade

## Abstract

Recently, accumulating evidence has elucidated the important role of T cells with stem-like characteristics in long-term maintenance of T cell responses and better patient outcomes after immunotherapy. The fate of T_SL_ cells has been correlated with many physiological and pathological human processes. In this review, we described present advances demonstrating that stem-like T (T_SL)_ cells are central players in human health and disease. We interpreted the evolutionary characteristics, mechanism and functions of T_SL_ cells. Moreover, we discuss the import role of distinct niches and how they affect the stemness of T_SL_ cells. Furthermore, we also outlined currently available strategies to generate T_SL_ cells and associated affecting factors. Moreover, we summarized implication of T_SL_ cells in therapies in two areas: stemness enhancement for vaccines, ICB, and adoptive T cell therapies, and stemness disruption for autoimmune disorders.

## Introduction

Physiologically, stem cells are responsible for tissue regeneration (1). They exist in multiple tissues, such as skin (2), cardiac muscle (3), lung (4), central nervous systems (5), intestine and blood (6, 7). In tumor, cancer stem cells (CSCs) sustained the heterogeneity of tumor cell populations (8, 9). Since hematopoietic stem cells (HSCs) are insufficient to support the high turnover of antigen-specific T cell for long-lasting immunological memory, which persisting for a lifetime without re-exposing to the antigen stimulation (10, 11). In 2001, Fearon’s team proposed stem cell-like properties of T cells to maintain T cell memory (12). Like HSCs, memory T cells also show some stem-like features like asymmetric T lymphocyte division (ASD) (13, 14). Possibly, the T cell population is originated from memory stem T cells.

**Figure 1:**
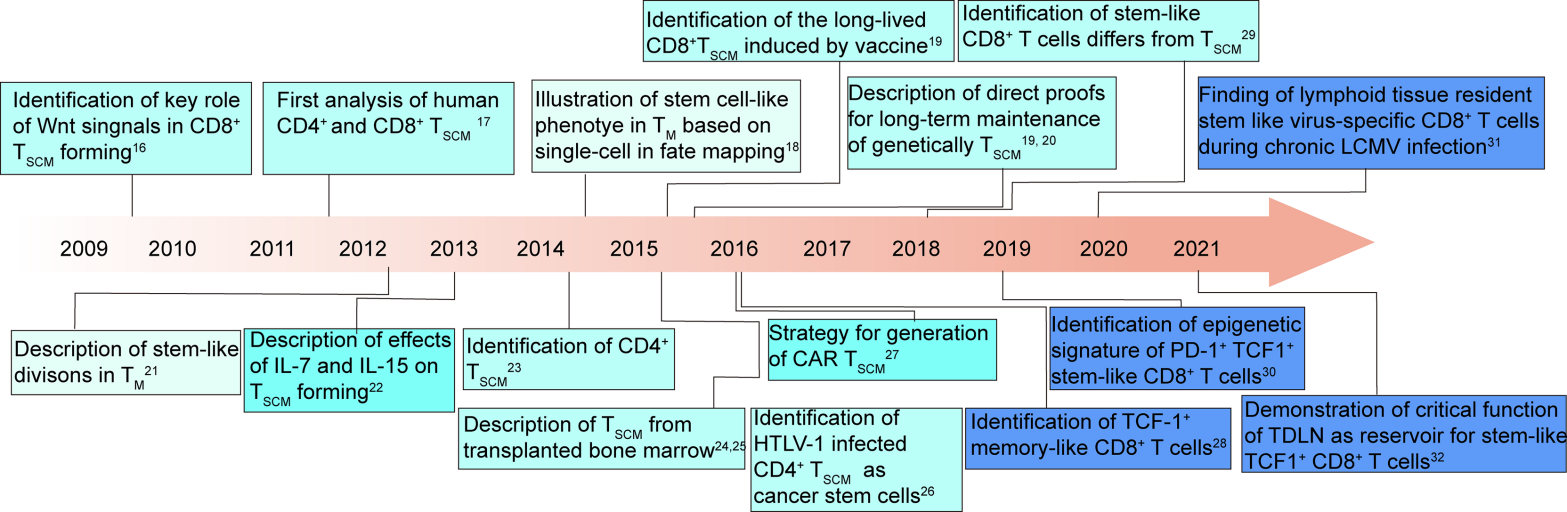
T cell stemness: milestones and key discoveries in the last 12 years (14, 16–31). T_M_, T memory cells; T_SCM_, T memory stem cells; HTLV-1, human T cell lymphotropic virus type 1; CAR, chimeric antigen receptor; LCMV, lymphocytic choriomeningitis virus; TDLN, tumor-draining lymph node.

Akin to stem cell in somatic tissues, these T cells generated and maintained T cell heterogeneity (14, 15), and shared a core transcriptional signature with HSCs (13). Recently, significant development can be seen in revealing the features and functions of stem-like T cells and how they can be harnessed to improve therapeutic outcomes (14, 16–31) (**Figure 1**).

The TCF1-positive stem-like T cells, differing from T_SCM_ cells, have been found in chronic immune model. These stem-like T cells are memory-like progenitor exhausted T cells. They are essential to maintain the immune response of T cell and determine therapeutic effects of PD-1 antibody (31).

Traditionally, niche is defined as a specific microenvironment that contains intrinsic an extrinsic cues for stemness retaining and continuous division (32).

Niche provides a spatial associations, which surrounded by extracellular matrix, cytokines and chemokines, which are produced by stroma, are essential for cellular homeostasis, self-renewal or differentiation (33). Integrins are essential to regulate the interaction of stem cell with surrounding niches, and mediate stem cell location and motion. Depending on contacting *via* integrin, stem cells receive cues from niches to sustain their activities (34–39). Bone marrow provides physical and chemical confinement to keep anatomic structure of the stem cell niches (40–42). But there is no direct evidence to prove that it similarly serves as a T_SCM_ cell niche.

Particularly, increasing evidence has shown that T cells need niches to maintain their long-lived memory and effectively respond to immunotherapy (40, 43). Further studies demonstrated that secondary lymphoid tissues (lymph nodes (LNs) and spleens etc.) provide homeostatic cues from fibroblastic reticular cell niches for T_SCM_ cells (44). These organs are specialized to support immune cells in antigen presentation, initial activation and proliferation (45, 46). In triple negative breast cancer (TNBC) patients, stem-like T lymphocytes against tumor have been found to residue in tumor-draining lymph nodes (TDLNs) (47). TDLNs, tertiary lymphoid structures and other intra-tumoral niches have been demonstrated to house CD8 positive stem-like T cells (48–50).

In this review, we describe emerging findings demonstrating the characteristics of subset of stem-like T (T_SL_) cells, mainly including stem-like memory T (T_SCM_) cells and stem-like progenitor exhausted T cells. We highlight how they exist and functionalize for human health and disease. We further demonstrate the potential signaling pathways that maintain stemness of T cells. Moreover, we discuss the import role of distinct niches and how they affect the stemness of T_SL_ cells. Furthermore, we also outlined currently available strategies to generate T_SL_ cells and associated affecting factors. Finally, we envision how to confer stem cell-like properties to T cells that might be used for immunotherapies.

Box 1 **Highlights**

This box includes main points of view of this review.

- Stem-like T cells (T_SC_) are a subpopulation of mature T cells that display the stem-like properties, maintaining long-lasting immune effect even among exhausting clones.
- All unique function of stem-cell like T cells is not completed proven by experiments. Further experimental evidence is still needed to comprehensively dig out all their unique function before an exact definition can be made.
- Secondary lymphoid tissues provide survival niches for most stem-like T cells of the adaptive immune system; besides, intratumoral APC niches and tumor-associated tertiary lymphoid structures also house stem-like tumor-specific lymphocytes.
- Approaches to generate stem-like T cells mainly include; pharmacological, cytokine and costimulatory signal-based regulation (e.g.GSK3β inhibitor, MEK inhibitor AKT inhibitor, IL-21, 4-1BB agonist, metabolic taming and TCR controlling), and gene-based regulation (T_SCM_-associated transcription factors or miRNAs, NOTCH signaling).
- Currently, clinical clinical-grade memory stem cells are mainly derived from naïve T cells culturing in with cytokines like IL-7, IL-21, and or IL-15, however, many other experimental methods are still not accessible in clinic.
- A significant number of researches have demonstrated that approaches to control stem-like T cells are necessary to achieve better outcomes in immune related diseases, such as HIV infection, Type I diabetes and cancer.
- Evidence from both experiments and clinical trials has demonstrated stem-like T cells determine therapeutic effect of immunotherapies, especially like immune checkpoint blockade (ICB), vaccines and adoptive cell therapy (ACT).

## Origin and hallmarks of stem-like T cells

### Origin of stem-like T cells

**Figure 2:**
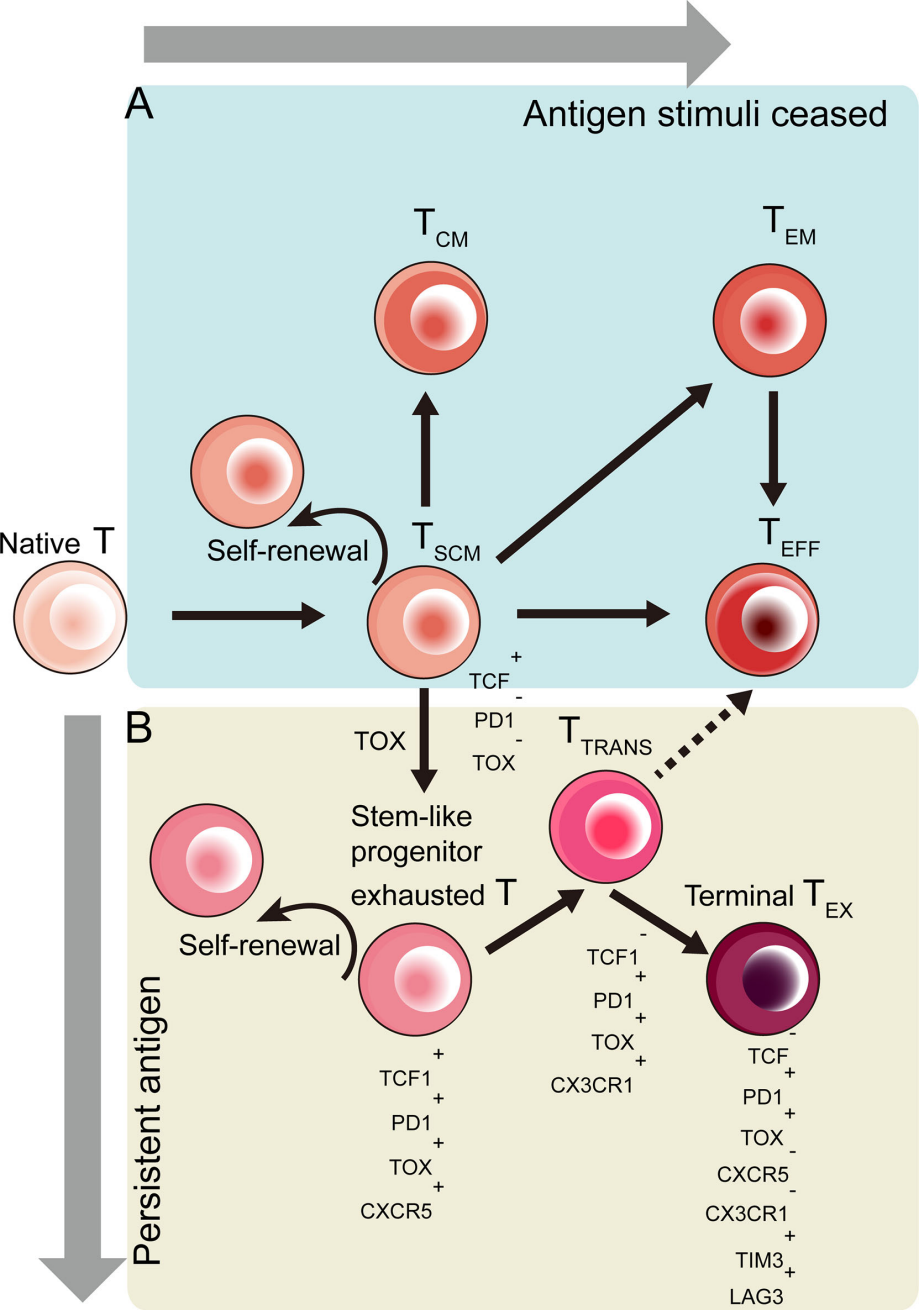
Developmental trajectory of CD8^+^ T cells toward effector (T_EFF_) and exhausted (T_EX_) CD8+ T cells in response to different antigen stimulation conditions. Stem-like T cells emerge during the T cell differentiation. **(A)** When antigen clear and stimuli cease, activated T cells differentiate into memory stem cell-like T cells (T_SCM_), central memory (T_CM_) T cells or effector memory (T_EM_) T cells in terms of signal strength (51). **(B)** Induced by TOX-based epigenetic regulation, T_SCM_ cells become stem-like progenitor exhausted T cells co-expressed TCF1, PD1, TOX and CXCR5. These stem-like exhausted T cells then become TCF1^-^PD1^+^TOX^+^CX3CR1^+^ transitory exhausted T (T_TRANS_) cells, which give rise to terminally exhausted (T_EX_) T cells (52–56).

Stem-like T cells (T_SC_) are a subpopulation of mature T cells that own self-renewal potential and can generate progenies by asymmetric division. These stem-like T cells exist in differentiating and even in exhausting process (**Figure 2**). The strength and duration of stimulatory signals decides the fate of naïve T cells: transform to Effector (T_EFF_) or exhausted (T_EX_) T cells.

Responding to antigen stimuli, naïve T cells differentiated into memory T cells, like central memory T (T_CM_) cells and effector memory (T_EM_) T cells and terminally, into T_EFF_ cells (51) (**Figure 2A**). The CD62L^+^ CCR7^+^ T_CM_ cells were considered to display stem cell-like phenotypes and functions among all memory T cells (12). Later in mice model, a new group of memory T cells were discovered with more stem cell-like attributes in comparison with traditional T_CM_ cells. Similar to naive T cells, these memory T cells have a similar CD44_low_CD62L_high_ phenotype. Moreover, they are a new Sca-1^+^ Bcl-2^+^ IL-2Rβ^+^ CXCR3^+^ T cells subset (16, 57). These T cells were named memory stem T (T_SCM_) cells.

CD8^+^ T_SCM_ cells are also found in humans, which highly express c-KIT indicating stemness, expel cellular toxins by ABCB1 (58). More recently, other studies have demonstrated highly expressed β-catenin as molecular clue to human CD8^+^ T_SCM_ cells (16, 59). Therefore, T_SCM_ cells can be regarded as stem-like memory T cells. Upon acute antigen stimulation, naïve T cells may differentiate into T_SCM_ cells, T_CM_ cells or T_EM_ cells after antigen clear. In virus infection condition, T_SCM_ cells, T_CM_ cells and T_EM_ cells can be found in latent or resolved infection (60).

However, primed T cells would differentiate into terminal T exhausted (T_EX_) cells upon chronical antigen stimulation (52). The exhausted PD-1 positive T cells are divided by differentiation hierarchy marked by their PD-1 expressing level, including less-differentiated PD-1^intermediate^ T cell that are able to respond to PD-1 blockade, and terminally exhausted PD-1 high T cells, which have inefficient function (27, 53, 61). Jolanda Brummelman et al. conducted scRNA-seq and found that these stem-like T cells do not exhibit typical properties as seen in circulating T_SCM_ cells (28).

These stem-like CD8^+^ T cells, which undergo TOX-driven epigenetic changes, are progenitor exhausted T cells co-expressing TCF1,PD1,TOX and CXCR5 (52–56) (**Figure 2B**).

Indeed, these stem-like CD8^+^ T cells are subset of exhausted T cells. But they differ from exhausted T cells by the expression of TCF-1 Under chronic antigen stimulation, Tex cell population can be generated independently of TCF-1-expressing Tpex cells, but they would be replaced by new T_ex_ cells derived from TCF-1^+^exhausted T cells progenitor T cells, which continuously activated, proliferated and differentiated into Tex cells (60). Some researchers demonstrated the development relationship among different exhausted T cells (62, 63). There are four subsets of exhausted T cells distinguished by expression of Slamf6 and CD69, and the four subsets were defined as progenitor 1 (Slamf6^+^CD69^+^; T_ex_^prog1^), progenitor 2 (Slamf6^+^CD69^-^; T_ex_^prog2^), intermediate (Slamf6^-^CD69^-^;T_ex_^int^), and terminal (Slamf6^-^CD69^+^; T_ex_^term^).Among them, the T_ex_^prog1^ cells are stem-like progenitor exhausted T cells we mentioned above, which had potential to generate all other three subsets of exhausted T cells. T_ex_ cells transited from T_ex_^prog1^ to T_ex_^prog2^ to T_ex_^int^ and to T_ex_^term^,accompanied by changes of TCF1,TOX, T-bet and Eomes expressions (from TCF1^hi^Tox^hi^ to TCF1^int^Tox^hi^ to TCF1^neg^T-bet^hi^Tox^int^ and finally to TCF1^neg^T-bet^lo^Tox^hi^Eomes^hi^). Further, Yao et al. emphasized the role of transcriptional repressor BACH2 for stem-like T cells to maintain their stem-like feature (64). But the mechanism about how stem-like cells prevent terminally exhausted cell fate is not well understand till now. There is still much to do to address this issue.

### The hallmarks of stem-like T cells

#### T_SCM_ Cells

According to Lugli’s depiction (65), T_SCM_ cells are generated with stem-like proprerties like strong proliferation potential and multipotency under healthy homeostatic conditions. These cells can rapidly generate progeny that produce granzyme and other cytokines. Besides, they express naïve T markers-CD62L and CCR7, and self-renewal markers- TCF1 and LEF1.

Stem-like T cells exhibit long-term persistence and the ability to renew to generate more differentiated cells. To monitor the persistence of these cells, genetically engineered T cells have been infused into the host, in order to easily trace these infused cells as antigen-experienced cells over time. Based on this approach, T_SCM_ cells have demonstrated decades of persistence in patients who suffer severe combined immunodeficiency (adenosine deaminase -deficient form) (20). Further studies demonstrated a strong correlation between T_SCM_ cell numbers and enhance d immune reconstitution capacity and longevity in hematopoietic stem cell transplantation (HSCT) models and chimeric antigen receptor T (CAR-T) cell therapy (66, 67).

To track individual transgenic T-cell clones, TCR-α/TCR-β labeling and viral integration can be used to classify according to the patient’s infusion product and T-cell differentiation phenotype during long-term follow-up. Similarly, in patients infected with simian immunodeficiency virus (SIV), T_SCM_ cells can be tracked for up to 70 days at high levels after infection (68). In another study, the number of T_SCM_ cell types gradually increased in HIV-1 patients receiving antiretroviral therapy (ART). After treatment, terminally differentiated effector (T_TE_) cells shrank more than T_EM_ cells and T_SCM_ cells (19). After all, these studies prove that human T_SCM_ cells have long-term self-renewal capacity and pluripotency.

Another feature of stem-like T cells is that they are also able to produce more differentiated progenies (69). Upon acute antigenic stimulation, T_SCM_ cells directly transformed from naive T cells and further differentiated into T_CM_, T_EM_, and terminally differentiated effector T cells (23, 70–72). Pais Ferreira et al. found that stem cell-like TCF7^high^ CD8^+^ T cells possess a central memory function and can quantitatively generate T_CM_ cells (73). In fact, only naive T cells and T_SCM_ cells were able to produce all memory T cell subsets (17, 19, 21, 51). Therefore, T_SCM_ cells are a memory T cell subset sharing some phenotypes with naïve T cells.

#### Identification of T_SCM_ cells from other T cells subset

Gattinoni’s group have been done a serial of studies to generate and identify T_SCM_ cells (16, 17, 74, 75). They illustrated that T_SCM_ cells are genetically similar to naïve T cells, showing only 75 differently expressed genes compared with naïve T cells. But they differed from naïve T cells by expression of CD95 and IL-2Rβ, and performing memory T cells functions, including low levels of T cell receptor rearrangement excision circles, replicative history, rapid acquisition of effector functions (IFN-γ, IL-2, and TNF-α production) upon antigen re-challenge. On the other hand, T_SCM_ cells differ also differed from conventional memory T cells with enhanced self-renewal and multipotency.

There were more T_SCM_ cells retained their input phenotype than T_CM_ cells did, indicating enhanced self-renewal capacity of T_SCM_ cells. Researchers have demonstrated increased fraction of effector functional T cells from T_N_ cells to T_SCM_ cells to T_CM_ cells to T_EM_ cells (17). Besides, T_SCM_ cells could generate all conventional memory T cells, including T_CM_ cells and T_EM_ cells. On the contrary, T_CM_ cell could not generate T_SCM_ cells. They further testified increased proliferation and survival of T_SCM_ cells compared with naïve T, T_CM_ and T_EM_ cells *in vivo*. Verma et al. also confirmed that T_SCM_ cells were distinct from T_CM_ and T naive cells and possessed the characteristics of an intermediated state between naïve T and T_CM_ cells (76). They demonstrated these T_SCM_ cells had high FAO-mediated metabolic fitness, self-renewability, high proliferation, multipotent capacity and antigen recall responses. These T_SCM_ cells are different from naïve T cells in capacity of antigen recall response. In other words, T_SCM_ cells are cover function of both naïve T and T_CM_ cells. Based on their findings that T_SCM_ cells owned a higher degree of unmethylated CpG sites at Tcf7 and methylated CpG sites at Ifng and Prf1 sites, it can be reasonably inferred that more T_SCM_ cells may be superior to T_CM_ cells in stemness when compared with T_CM_ cells.

Indeed, existing observations have provided some experimental evidence for specificity of stem-like T cells among all T cells. However, it may be not sufficient to completely isolate this subset of T cells out of memory T cells and defined as T stem cells. All unique function of stem-cell like T cells is not completed proven by experiments. Further experimental evidence is still needed to comprehensively dig out all their unique function before an exact definition can be made.

#### Stem-like progenitor exhausted T (T_pex_) cells

According to Lugli’s depiction (65), T_SCM_ cells are generated with stem-like proprerties like strong proliferation potential and multipotency under healthy homeostatic conditions or acute antigen stimulation. While TSL cells herein specifically refers to exhausted precursor T cells with stem-like properties under the condition of chronic antigenic stimulation. Therefore, compared with T_SCM_ cells, T_SL_s are a group of cells that are closer to exhausted T cells, and thus have certain characteristics of exhausted cells including increased expression of PD-1 and decreased secretion of TNF-α and IFN-γ (65).

TCF1 is the key factor to determine the recall expansion and self-renewal of stem-like PD-1^+^ T cells (77). These cells also express TIGIT, but not other negative regulators. Furthermore, sustained TCR stimulation is needed for the differentiation into terminal effector or exhausted T cells. Besides, conventional type 1 dendritic cells, CD28 co-stimulation signals, and the cytokine IL-12 in tumors are needed for TIL re-activation under treatment of immune checkpoint blockade, suggesting that these factors are key regulators for PD-1^+^ TCF1^+^ progenitor TILs. Besides, IL-27, c-MAF, PRDM1, NR4A1 and TOX support the immune effect and inhibit potential. However, how these factors affect less-differentiated PD-1^+^ TCF1^+^ T cells versus PD-1^+^ TCF1^-^ T cells should be directly evaluated. During T cell priming, the expression level of TCF1 decides the fate of CD8^+^ T cells: enter into memory T cell pool or differentiated effector cell pool (16, 56, 73, 78–91).

CXCR5^+^exhausted CD8^+^ T cells play a key role in controlling replication of virus in lymphocytic choriomeningitis virus (LCMV)-infected mice (61). These CXCR5^+^ PD-1^lo^ TIM-3^lo^ KLRG1^hi^ T cells are less exhausted than their CXCR5^-^ counterparts. This result suggests that CXCR5^+^ KLRG1^+^CD8^+^ PD-1^lo^ TIM-3^lo^ T cells may have stem cell-like properties. Based on high-dimensional single-cell analysis, additional studies demonstrated that these T cells retained signal networks regulating stem cell-like properties and functions (28). Similarly, other chronic viral infection studies also discovered CXCR5^+^TCF1^+^PD1^+^ TIM3^-^ T cells with stem-like features and CXCR5^−^TCF1^-^ PD1^+^ TIM3^+^ T cells with terminally differentiated features (53, 54, 56). Therefore, CXCR5 may be another key marker for stem-like progenitor exhausted T cells.

Galletti’s group had illustrated difference between fwo subsets of stem-like CD8+ memory T cells progenitors (PD1^-^TIGIT^-^ subsets versus PD1^+^TIGIT^+^ subsets). PD1^-^TIGIT^-^ subsets were committed to functional progeny, while PD1^+^TIGIT^+^ subsets were committed to dysfunctional progeny (82). Acccording to their data about phenotype and transcription charaters, these two subsets correspond to T_SCM_ cells and T_SL_ progenitor exhausted cells as we described in this paper. In order to fast distinguish T_SL_ progenitor exhausted cells and T_SCM_ cells, we summarized serval key difference of them in **Table 1** (60, 65). Besides, as T_SCM_ cells are functionally similar to T_CM_ cells, we also compared T_SCM_ and T_CM_ cells together in this **Table 1**. As shown in this table, T_SL_ progenitor exhausted cells can be distinguished from T_SCM_ cells mainly by the generation condition. T_SCM_ cells were generated under physiology stimulation or acute antigen stimulation, while T_SL_ progenitor exhausted cells. Besides,T_SL_ cells cannot perform high proliferation unless treated by immune checkpoint blockade. Besides, the effector progency derived from T_SL_ progenitor exhausted cells cannot produce Granzyme and express high level of PD1. Additionally, T_SL_ exhausted progenitor cells maintained their survial independent of CD4^+^ T cell help, while T_SCM_ cells needed the help of CD4^+^ T cells. On the other hand, although T_SCM_ cells possessed most function that T_CM_ cells also have, they can be differentiated from T_CM_ cells by enhanced stem-like properoties, such as proliferation potential. In addition, T_SCM_ cells are always CD45RA-positive, while T_CM_ cells are CD45RA-positive. 

**Table 1:** Comparison between T_SCM_,T_CM_ and stem-like progenitor T_EX_ cells (60, 65).

Conditions			
Physiology stimulation Stimulation	Names		
		T_SCM_ cells	T_CM_
	Proliferation potential	+++	++
	Key markers	CD45RA^+^CD62L^+^CCR7^+^CD27^+^CD95^+^	CD45RA^-^CD62L^+^CCR7^+^CD27^+^CD95^+^
	Function	Re-elicit effector function: granzyme (GZMB) and cytokine production; Dependent of CD4^+^T cells help	Re-elicit effector function: granzyme (GZMB) and cytokine production; Dependent of CD4^+^T cells help
Chronic stimulation		T_SL_ progenitor exhausted cells	
	Proliferation potential	Unleashed upon immune checkpoint blockade (ICB)	
	Key markers	TOX^+^CCR7^+^CXCR5^+^TIM3^-^	
	Function	Re-elicit effector function:mixed phenotype of effector cells (CCR7^-^GZMB^-^) and exhausted cells(PD1^+^); Dependent of CD4^+^T cells help

## Effects of stem-like T cells on human health and disease

### Negative effects

When T_SCM_ cells are abnormal and self-reactive, these T cells may cause pathogenic effects (83). For example, CD4^+^ T_SCM_ cells may support viral replication and transcriptionally silenced forms of infection in HIV-1 infections (84). Furthermore, HIV-1 is able to use CD4^+^ T_SCM_ cells as an extremely durable, self-renewing viral reservoir that persists despite antiretroviral therapy for up to approximately 277 months (85). Similar conclusion is reached in patients with HTLV-1 infection. HTLV-1 infected CD4^+^ T_SCM_ cells can act as a cancer stem cells contributing to the spread and preservation of malignant cells infected by HTLV-1.

T_SCM_ cells also exhibit negative effects in autoimmunity. Aplastic anemia is regulated by autoreactive CTLs that target hematopoietic progenitor cells. Compared with healthy people, patients with aplastic anemia have increased frequency and activation status of CD8^+^ T_SCM_ cells. Furthermore, the growing number of CD8 positive T_SCM_ cells after immunosuppressive therapy indicates bad prognosis (86). A recent genome-wide association study further pinpoints the effect of CD4^+^ T_SCM_ cells in the autoimmune and other lymphoid diseases. It indicated that the number of CD4^+^ T_SCM_ cells indicates those susceptible individuals to juvenile idiopathic arthritis or chronic lymphocytic leukemia (87). Till now, it can be hypothesized that self-renewal autoreactive T_SCM_ cells may lead to long-lasting inflammatory responses, which may explain the long persistence of these diseases. But how T_SCM_ cells affect autoimmune disease must be investigated in dedicated studies. In general, T_SCM_ cells, especially CD4^+^ T_SCM_ cells, play negative role in many virus-induced diseases and autoimmune diseases. However, how T_SCM_ cells act in diseases like type 1 diabetes, thyroiditis and autoimmune hepatitis is currently poorly understood.

### Positive effects

T_SCM_ cells are vital in maintaining long-term protection in many acute and chronic infections (17, 19, 88, 89). However, how these T_SCM_ cells generate during immune response remains unclear. It is difficult to dynamically study T cell activities in human by the limitation of knowing exact timing of infection. Active vaccination, especially smallpox and yellow fever vaccines, offer the possibility to induce an immune response in a supervised manner, are particularly suitable models for primary acute viral infection in humans (90). Based on this, further study has been made to entirely uncover how T_SCM_ cell form and maintain for a long time by using the YF vaccine as a model system (19). In line with results on SIV infection in NHP (68), YF-specific T_SCM_ cells existed early after receiving vaccine, and persisted at stable levels for decades (19). The frequency of T_SCM_ cells are responsible for continuously replenishing exhausted effector T cells, so that to control persisting infections (19, 27, 55, 91).

Notably, some researches reveal that T_SCM_ cells are unable to show positive effects in chronic viral and parasitic infections (88, 89, 92). However, T_SCM_ cells are generally necessary for long-term immune effect acute and chronic microbial infections. The existence of SIV and HIV-1 infected T_SCM_ cells is closely correlated to the development of symptomatic immune deficiency after virus infections (93, 94). In fact, SIV DNA copies were found in CD4-positive T_SCM_ cells of rhesus monkeys, which normally exhibit AIDS-like clinical manifestations when untreated. But in SIV-infected CD4^+^ T_SCM_ cells of sooty mangoes (a group of NHPs), SIV DNAs are not found. This group is more likely to be asymptomatic carriers even when large number of virus can be detected in circulation system (95, 96). Similar results are also found in viremic nonprogressors, who have less HIV-1 DNA carried CD4^+^ T_SCM_ cells than ordinary patients. Despite their low frequency, T_SCM_ cells are pivotal to produce the circulating CAR T cell pools substantially, and determine early anti-leukemic responses in patients (97).

Current vaccines are often not efficient enough to induce robust and long-term immunological effect, because they mainly focus on induce CD8^+^ T_EM_ cells other than T_SCM_ cells (98, 99). In fact, these T cell vaccines are inferior to vaccines that can induce protective antibodies (100, 101). In order to improve efficiency of T cell vaccines, methods should be developed considering induced stem or memory T cell pool at gene, transcription or metabolism levels (102).

Indeed, small number of T_SCM_ cells can be induced after natural infections or administration of cancer vaccine, despite that effector cells are initially predominant cell subsets (103). On the other hand, T_EM_ and tissue-resident memory cells should be reserved to guarantee immediate protection against re-infection (104–106). Altogether, ideal vaccines should be designed to reconstitute entire diversity of memory T cell subsets *in vivo* (107, 108).

## Stem-like T cells and niches

### Niches in bone marrow

In adult immune system, T_CM_ cell pool has been considered as one cell subset with stem cell characteristics (16, 109). Bone marrow is crucial in sustaining the persistence of memory T cells (40, 41). However, it is unclear if the bone marrow plays role to support T_SCM_ cell as their niche.

Memory T cells were previously thought to share some attributes with p HSCs (13). Further, the existence of two distinct BM niches, including quiescence niches and self- renewal niches, was found to be a main reservoir for memory T cells. Together with similar niches in other organs, BM niches maintain the pool of memory T cell (12, 110). Indeed, bone marrow provides pivotal cues, such as IL-7, to support long-lasting pathogenic CD4^+^ T cells (111). Most dividing T cells reside in BM other than in spleen or LNs, despite that only less than 2% memory CD8^+^T cells in the BM enter into proliferating cell phase (112, 113). In bone marrow, such dividing is antigen-independent and equality occurs among CD8^+^T cells that had experimentally primed long before or those not express antigen before (113, 114).

Notably, there are approximately 2% of the cells are cycling, and BM memory CD8^+^T cell in BM affect state of HSCs (115). Both HSCs memory CD8^+^T cells are regulated by two kinds of BM niches: quiescence and self- renewal niches. Under physiological conditions, quiescence niches not self- renewal niches are predominant niches for both HSCs and memory CD8+T cells. But in some diseases, like BM T cell cancer, self-renewal niches might grow (116). Memory phenotype cells may include both antigen-reactive T cells and bystander T cells (117). The frequency of these two groups of memory T cells changes according microenvironment. Based on this, someone observed different Ki67 staining results in animals-primates and mice, living in living in normal (118) or SPF environment (41).

In fact, the majority of memory T cells in bone morrow enter into the periapical tissue and are replenished by newly coming memory T cells. This conclusion was reached by many researches through methods including bromodeoxyuridine (BrdU) pulse-tracking (110), *in situ* labeling (119), and parabiosis experiment (120).

Taking together, the two niches in the BM not only maintain sustained recirculating memory T cells for re-circulation without antigen stimulation, but also quickly regulate division and migration of these cells when encounter disturbing (121). Upon perturbation, the number of BM niches grow to produce more relevant factors, which not only support T cell survival and growth but also attract more memory T cells into the self-renewal niches. Of note, the BM CD8^+^T cell proliferate in response to stimulators that boost innate immune effect. Someone found that polyI:C alone could evoke proliferation of memory CD8^+^T cell *in vivo*, particularly in the BM (122).

### Niches in node

Connolly et al. found that T cells in tumor-draining lymph nodes (TDLNs) may be precursors of TCF1^+^ T cells and responsible for persistence of T cell responses in lung cancer. Therefore, TDLNs serve as reservoirs for TCF1^+^ tumor-specific CD8^+^ T cells throughout development (31). Similarly in triple negative breast cancer, Researchers demonstrated tumor-draining LNs (TDLN) worked as niches for CD8^+^T lymphocytes against lymph-draining antigen (48).

**Figure 3:**
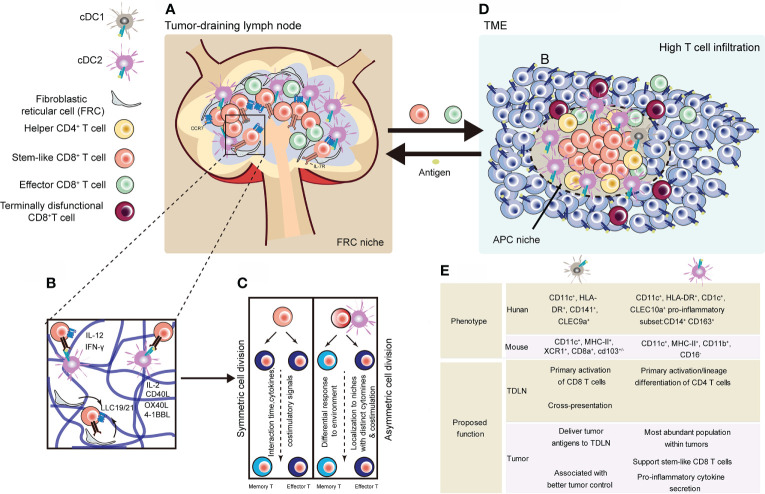
Representative niches supporting the maintenance of stem-like CD8^+^ T cells. **(A)** Stem-like CD8^+^ T cells in lymphoid niches differentiate into cytotoxic effector T cells, regulating tumor regression (44). **(B)** CCR7^+^ naïve CD8^+^ T cells localize and in the inner cortex (blue) and depend on homeostatic factors from niche supported by fibroblastic reticular cell. Activation process of CD8^+^ T cells can be divided into three phases, during which regulation signals are unleashed from surrounding, especially DCs (123–125). **(C)** After activation signals, T cells undergo symmetric or asymmetric cell division. Daughter cells transform into TEFF or memory T cells depending on their distance to DCs (32, 126). **(D)** Tumors with high dense of TILs show APC-niches, containing cDC1, cDC2 and helper CD4 T cells and stem-like CD8 T cells (50, 127–130). **(E)** Comparisons of human and mouse cDCs found in TDLNs and tumors (43, 49, 50, 127, 131–134). cDC, conventinal dendritic cell; TDLN, tumor-draining lymph node (129, 130, 135–140).

T_SCM_ cell homeostasis may depend on cues from FRC-based lymphatic niches (44) (**Figures 3A–C**). Lymph nodes (LNs) depend on a number of stromal cells, such as fibroblastic reticular cells (FRCs), to build lymphatic macroniches and guide cell-cell interactions (141). These cells provide integrin, chemokine, and cytokine cues to keep multipotency of lymphocytes, while also give rise to differentiated cells upon pathogenic challenge. Therefore, lymphatic niches must maintain homeostasis so that to avoid inappropriate immune responses and autoimmunity.

T cells mainly locate in cortex (including outer cortex and inner cortex) of LNs (141). The outer cortex is rich in CD4^+^ follicular T helper cells, while the inner cortex, also known as the T cell zone, is rich in both CD4^+^ and CD8^+^ T cells. Other important cells in T cell zone include dendritic cells (DCs), and fibroblastic reticular cells (FRCs) (142).

Medulla is innermost region, containing plasma cells and macrophages (143). Specific receptors and ligands in niches are important for niche development and maintenance. Once these receptors and ligands were knockout, it may change lymph node structure and alter cell localization and responses (144–146). Numerous key studies describe cell-cell interaction and their behavior in lymph nodes upon immune stimulation (147–150). In the paracortical macroniche, CCL21/19 secreted by FRC regulate the movement of DCs and T cells in FRC network, and FRC also produces survival and proliferation factors such as IL-7 and IL-15 (151–153). The process of T cell activation can be divided into three distinct phases according to T cells migration patterns along the FRC network (154).

The fate of CD8^+^ T cell may be conferred by T-APC interactions during early T cell activation (**Figure 3B**). APCs can generate inflammatory factors like IL-12, IFN-γ, and IFNαα, which control function and activities of T cells (155). In the third phase, CD8^+^ T cells transiently contact with APCs and received additional signals such as IL-2, CD40, CD27, 4-1BB, OX40, and TNFR2 (123–125). After activation signals, T cells undergo symmetric or asymmetric cell division (126) (**Figure 3C**). Moreover, the fate of T cells is tuned by CXCR3-mediated signals. Based on CXCR3-CXCL9 axis, T cells move out of center of lymph node and lose some stem-like memory features (131).

### Niches in tumor

Intratumoral tertiary lymphoid structures (TLSs) have been found in breast, colorectal, lung, hepatocellular and pancreatic cancers. TLSs might form through IL-22-, IL-23-, CXCL13-, CXCL19-, CXCL21- and LTα1β2-mediated signals (132). TLSs can be considered small ectopic lymphoid structures found within the tumor stroma. They also contain both B cell zone and T cell zone. TLSs may promote local antitumor immune responses, because TLSs can be linked to higher CD8^+^ T cell infiltration. Recently, several studies identified positive correlation of B cells found in TLSs with good outcomes of ICB treatment in patients (43, 133).

Contrary to tumors in absence of TLSs, metastatic melanoma tumors that own abundant TLS have larger number of less differentiated TCF1^+^ T cells (49). Dense APC niches were found in patients with kidney cancer. These niches keep phenotype of TCF-1 positive T cells in tumor (123). Jansen et al. also discovered dense antigen-presenting-cell niches within tumor to keep stem-like T cells, which support extensively infiltrating of T cells (50).

Although further researches are needed to uncover the role of these structures for effect on immunotherapeutic outcomes and tumor-specific CD8^+^ T cell function, it can be inferred that TLSs and APC niches are predictive markers of immune checkpoint blockade (ICB). Additionally, it will be important to develop immune-monitoring platforms to visualize the immune response organization inside the tumor before or during ICB therapy (134).

Both CD4^+^ T cells and DCs play important role in intratumoral niches to facilitate the response of CD8 T cells (50, 127–130) (**Figure 3D**). These intratumoral niches are immune cell rich structures, where stem-like CD8 T cells locate). DCs are mainly composed of Conventional dendritic (cDC) 1 cells (cDC1s) and cDC2s are two main types of DCs. cDC1s have been widely investigated in many kinds of cancer based on the fact that they can crossly present exogenous antigens and are prodominately APCs to prime CD8^+^ T cells (135, 136). Their function has been studied in many tumor models, as they are able to improve anti-tumor effect of CD8^+^T cells (129, 130). It has also been demonstrated that CCR7 promotes migration of cDC1s into TDLNs, where they present tumor antigen to CD8^+^ T cells (137). Resonating these results, other studies in human also observed that increased cDC1 associates with better overall survival in patients with cancers (129). However, the proportion of cDC1s is much lower than that of cDC2s within tumors (136, 138). And cDC2s preferentially perform as CD4^+^ T cells initial activator relying on MHC class II (MHC-II)-antigen complex (138). A recent study revealed that DC2s are a heterogenous population and can gain different properties (139). But their role in the tumor response remains unclear. A recent study focused on the interaction of cDC2s and CD8^+^ T cells within breast cancer. They found the presence of CD14-expressing cDC2 in tumors correlates with infiltration by tissue-resident CD8^+^ cells (140). Thus, cDC1s may differ from cDC2s in their roles for activating and differentiating antigen-specific CD8^+^ T cells in tumor. cDC1 population may mainly play its major role in TDLN, whereas the cDC2 population may maintain intratumoral CD8^+^ T cell responses. The different types of cDCs and their potential roles in the antitumor response are summed up in **Figure 3E** (129, 130, 135–140). Together, the presence of APC-rich niches and other intratumoral lymphoid structures (e.g. TLSs) may be an explanation of long-lasting T cell immune response in chronic antigen stimulation.

## Mechanisms involved in T cell stemness

### Asymmetric T lymphocyte division (ACD)

Asymmetric T lymphocyte division (ACD) ACD is speculated to be a mechanism for CD8^+^ memory T cell development (126). Stem-like memory cells are derived from one subset of daughter cells arising from ACD. Besides, T_CM_ cells arising from ACD have ability to replenish the CD8^+^ effector T cells population in response to antigen exposure (16, 109). Driven by IL-2Rα and T‐BET, T_CM_ cells differentiated into effector T cells (156, 157). T cells experience an effector fate resulting from the asymmetric segregation. Similar to other stem cells, T_SCM_ cells rely on activity of telomerase to keep telomere length and replicative ability (158, 159). Thus, ACD can be considered as a conserved mechanism giving rise to a subset of stem-cell-like, less-differentiated memory precursor cells during antigen stimulation (32).

### Master regulators of stemness

Upregulation of TCF-1 in follicular helper T (T_FH_) cell may induce expression of CXCR5 and PD-1. Meanwhile, TCF-1 enhances expression of BCL6, and further promotes early differentiation (160, 161). TCF-1 further represses expression of Blimp1 and IL2Ra, restricting differentiation toward TH1 pool (162, 163). Moreover, TCF-1 increases IL-6 responsiveness by its enrichment at the IL-6 receptor gene locus. LEF-1 is also a key regulator of T cells stemness, Both TCF-1 and LEF-1 is essential to control early development of T_FH_ cells (160, 164). Additionally, TCF-1 keeps the expression of Achaete-scute homologue-2 (ASCL2), which downregulates expression of CCR7, leading T cell migration to the border of T and B cell zone (165). Besides, TCF-1 also regulates TFH cell commitment and growth by targeting costimulatory marker inducible T cell co-stimulator (ICOS) (160). Among the TH cells subsets, T_FH_ cells are the main component to from memory pool to maintain stem-like properties against stress in chronic infection (166, 167). These cells help B cell helper re-initiate function (168). And T_FH_ stemness was kept in the presence of TCF-1 (169), which drives development of CD8^+^ central memory precursors by increasing expression of Eomes.

TCF-1 plays similar role and relies on similar mechanism in CD8^+^ T_SCM_ and T_FH_ cell differentiation to regulate cell stemness and memory (170). For example, TCF-1 represses Tbx21 and Prdm1 but induces Bcl6 expression in memory precursor CD8^+^ T cells. Further, TCF-1 is also a key player in regulating sustaining immune memory and preserving T cell stemness against chronic or acute infections (27, 75, 171). Loss of TCF-1 hinders the generation of stem-like progenitor cells pool in CD8^+^ T cells, resulting in rapid decrease of CD8^+^ T cells and viral control impairment (53, 75). Together, TCF-1 plays a shared role between T_FH_ and central memory T_SCM_ cells for suppressing exhaustion and immunologic recall response.

### Self-renewal pathways in T cells

**Figure 4:**
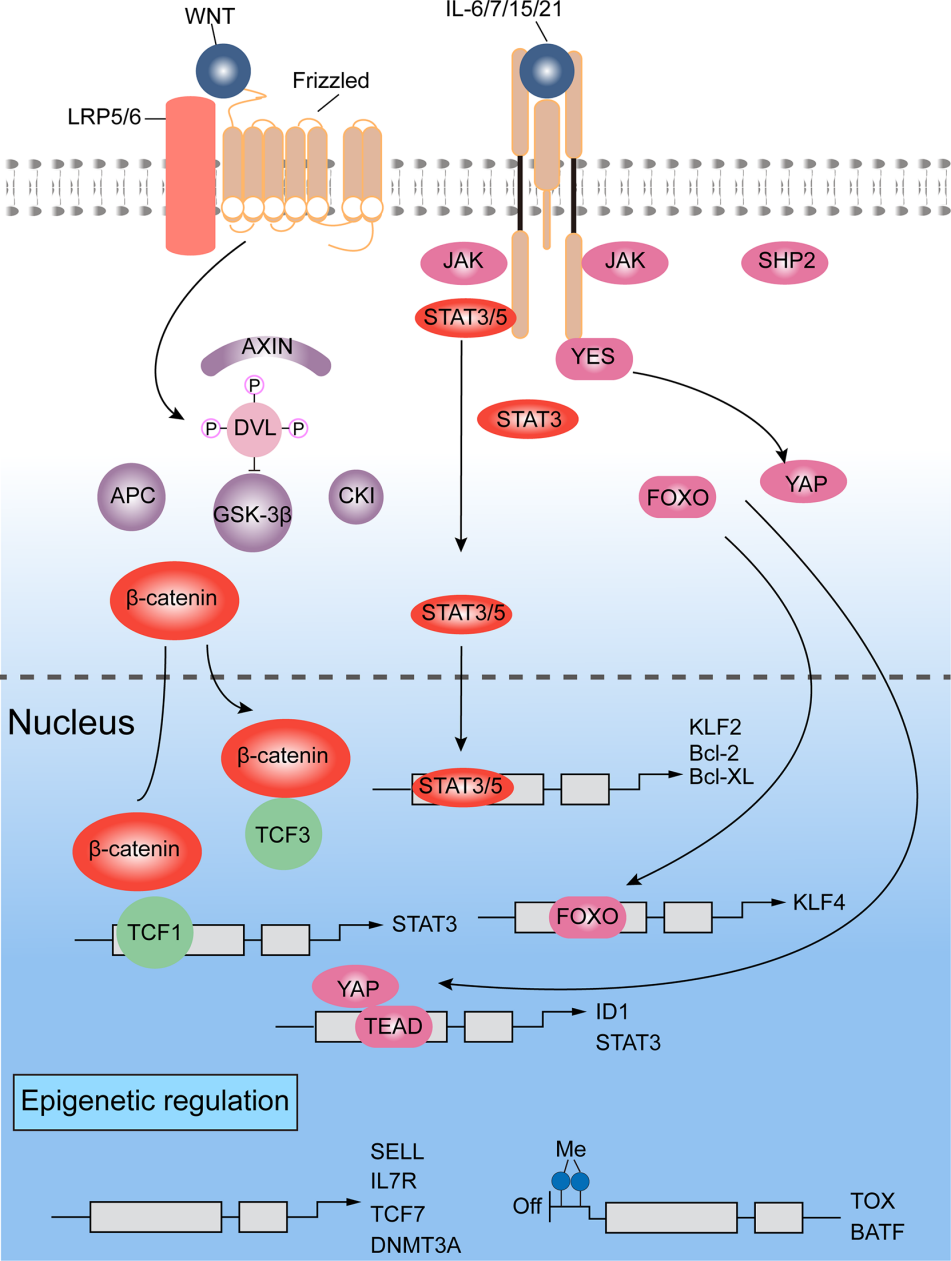
Signaling pathways and epigenetics regulating self-renewal of T lymphocytes (16, 80, 109, 172–181). WNT-β-catenin and transducer and activator of transcription 3 (STAT3) signals are mainly involved for the self-renewal regulation. Additionally, a crucial role is played by chromatin modifiers, which can add repressive histone marks to genes responsible for maintaining stemness, or catalyze DNA methylation on genes regulating differentiation.

A representative mechanism is shown in **Figure 4**, describing how T cells devote to self-renewal character and keep stemness on epigenetic level. In details, T cells also rely on WNT-β-catenin signaling to obtain stemness (**Figure 4**). Once WNT ligating to the Frizzled receptor and low-density lipoprotein receptor related proteins (LRP), Dishevelled protein (DVL) is activated to inhibit the formation of destruction complex (containing glycogen synthase kinase 3β (GSK3β) (172), which mediates degradation of β-catenin by proteasome. Then, β-catenin accumulates in cell nucleus, where it interacts with different kind of DNA-binding partners, such as members of the TCF and LEF family, leading chromatin remodeling and transcription modulating. TCF7 and LEF1, the WNT signaling transducers, which are found highly expressing in naïve T cells and CD8^+^ T_SCM_ cells and lost in more differentiated T cells, may maintain the stemness of T cells (16, 109, 173, 174). WNT3A or GSK3β inhibitors have been shown to promote the generation of self-renewing T_SCM_ and T_CM_ cells, and inhibit the progressive differentiation of naïve T cells (175, 176). In line in with these findings, stabilizing expression of β-catenin also inhibited the acquisition of effector T cell functions (177). In several studies involved in infection, memory T cells were increased by overexpression of TCF1 and β-catenin stabilization (178). Contrarily, it could be found that T_CM_ cells were depleted when TCF-1 was knocked out, which increased expressing levels of granzyme B and KLRG1 in T cells, but prevented the establishment of long-term T cell memory (80, 179). And further study revealed that TCF-1 regulate stemness depending on its binding capacity to β-catenin (80).

Interestingly, the center role of β-catenin in Wnt/β-catenin pathway for memory regulation of CD8^+^ T cell was challenged by some researches. Someone observed arrest of effector differentiation by GSK3β inhibitors even without β-catenin (175). In a mouse model, T cell memory and cell function were not found to be impaired by T cell-specific deletion (182). However, WNT reporter activity was not hindered by β-catenin deleting, which suggested that there is compensation mechanism for lack of β-catenin in WNT signals (183). Of note, a 52 kDa fragment of β‐catenin, which mediates interaction with TCF protein, retains some functionality (184). In addition, γ-catenin, which is also regulated by the destruction complex, could substitute β-catenin to promote transcriptional activity of TCF and LEF in cells showing β-catenin deficiency (185).

Other signaling involved in self-renewal of T cells include STAT3, SMAD and Yes-associated protein (YAP). KLF4 and KLF5 are the downstream genes of LIF-STAT3 signaling (186). On the other hand, direct activating complex of LIF receptor and GP130 recruit SHP2 to exciting MAPK signals. But this pathway is mainly for transmitting pro-differentiation cues rather than self-renewal signals (187, 188). Indeed, mature T lymphocytes can achieve long-term memory by triggering STAT3 activity based on environmental signals, which are triggered by IL-6, IL-7, IL-15 and IL-21. For instance, IL-21 suppresses forming of CD8^+^ T_EFF_ cells, maintaining T_SCM_ pool and supporting long-term T cell survival (189). STAT3 might also limit cell differentiation by activating KLF, which keep quiescence state of cells and induce lymphoid-homing molecules expressing (190, 191).

YAP has been found to enhance expression of ID protein based on BMP-SMAD pathway, and induce binding of LIF to the transcription factor TEA domain, resulting in expressing of pluripotent genes. This process could promote ESC self-renewal (192–194). As AKT/Hippo signals regulate YAP negatively, activation of Hippo pathways by cytokine IL-2 resulted in YAP degrading and differentiation-associated molecules expressing, further preventing CD8^+^ T cells senescence in response to viral infection (195). In ESCs, YAP may also enhance stemness associated transcriptional regulators, including STAT3 and members of ID family, but further studies are needed to confirm this mechanism in T cells.

### Epigenetic regulation

Once activated, the effector related genes of T_N_ CD8^+^ cells are epigenetically regulated before they gain memory and effector features. As such, CD8^+^ T_SCM_ cells show some effector features, while retain naive-associated transcriptional programs fundamental for self-renewal and for homing to lymphoid tissues (180). Therefore, epigenetic mechanisms are essential to regulate the differentiation of self-reactive CD8^+^ T cells. Epigenetic modifications not only regulate genes for silencing stemness, for example TCF7, SELL and CCR7, but also genes for memory and effector-associated factors (e.g. EOMES and TBX21) (180) (**Figure 4**). Furthermore, methylated modification happens on chromatin (like H3K9me3 and H3K27me3), resulting in suppression effect on gene expression (181).

By profiling genome-wide DNA methylation in normal versus autoimmune responses (196), Abdelsamed et al. built a whole epigenetic profile was made for entire human CD8^+^T cell population differentiation including T_N_, T_SCM_, T_CM_, T_EM_ and T_EFF_ cells. Based on this profile, the CD8^+^ T cell differentiation was further studied in type 1 diabetes exposed to chronic antigen stimulation. They discovered There were 3,000 most variable CpG loci found to distinguish different T cell subsets. In this way, T_SCM_ cells are found to be equally distant from both T_N_ and the T_EFF_ cells.

In fact, autoreactive CD8^+^ T cells and T_SCM_ cells show same methylated features on differentiation-associated genes like BATF, DNMT3A and TOX. TOX and BATF are responsible for CD8^+^ T cell exhaustion and effector function specification seperately (197, 198). These two factors are repressed by methylation in both T_SCM_ and beta cell specific CD8^+^T cells, while they are free from methylation marks in more differentiated CD8^+^ T cells. On the other hand, DNA methyltransferase DNMT3A, which involves in self-renewal regulation of stem cells, exhibits opposite methylation patterns. Of note, researchers found a self-reactive CD8^+^ T cell subset, which is clearly different from T_N_ and T_EFF_ cells, showing a mixed epigenetic pattern. Indeed, both stemness-associated-genes (such as LEF1, TCF7 and DNMT3A), and differentiation-involved-genes (such as TBX21, INFG, PRF1 and GZMK) opened, leading mixture of stem-like effector features in the same cells. Above studies suggested that progressive differentiation of CD8^+^ T cell is accompanied by great changes in epigenetic modification, degree of gene loci openness, and stem- and effector-like programming.

## Strategies to generate and investigate stem-like T cells

### Generation methods

**Figure 5:**
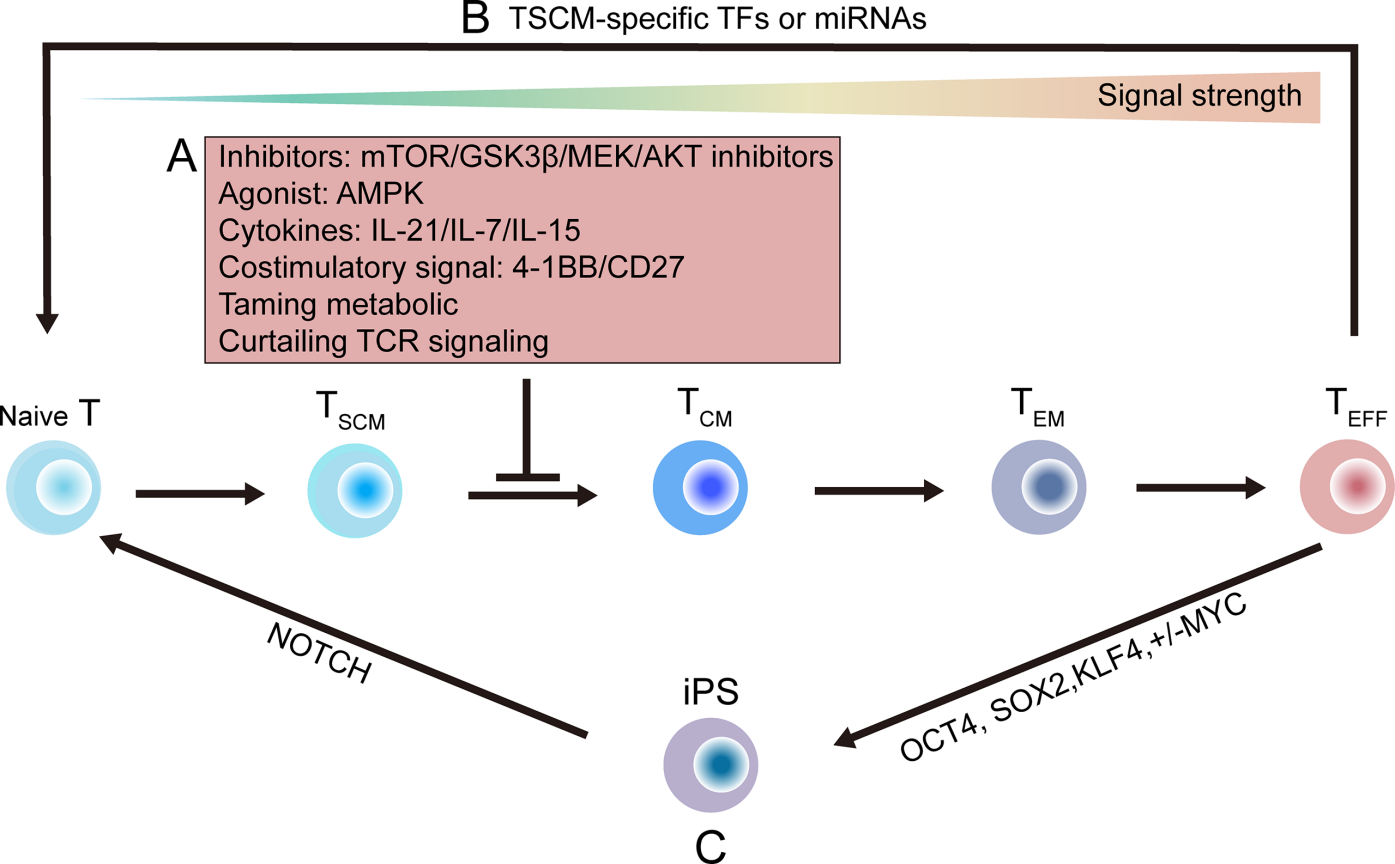
Strategies that might be utilized to generate stem-like T cells. **(A)** Progress of T cells differentiation toward effector T (T_EFF_) cell pool depends on the strength of stimulatory signals. Differentiating progress of primed T_N_ cells can be delayed or suppressed by targeted inhibitors (such as mTOR, GSK3β, MEK, AKT inhibitor); or by using cytokines, such as interleukin‐21 (IL-21), interleukin-7 (IL-7), interleukin-15 (IL-15), or by using proper costimulatory signal; or by taming metabolic modulators; or by curtailing TCR signaling (16, 21, 109, 201–204). **(B)** Direct reprogramming methods using naïve- or stem-associated factors or miRNAs (205, 206). **(C)** A two-step induction method is shown. By enforcing expression of OCT4, SOX2, KLF4 and MYC, terminal T_EFF_ cells can be programmed into iPS cells, which are then re-differentiated into TN cells by inducing NOTCH signals (207–214). GSK3β, glycogen synthase 3β; T_N_, naïve T; T_EFF_, T_CM_, effector T; iPS, induced pluripotent stem; OCT4, octamer-binding transcription factor 4; SOX2, sex determining region Y BOX 2; KLF4, Kruppel-like factor 4.

Multiple signal pathways, deriving from factors like TCRs, cytokines, co-stimulatory receptors and growth factor receptors, regulate the fate of effector and memory T cells (199–201). These signal pathways can be regulated by many small molecular drugs-mTOR inhibitor rapamycin (201), AMPK agonist metformin (202), GSK3β inhibitor (16, 109) and AKT inhibitor (203), can be quickly applied in new clinical trials. Success clinical practice have been released in solid organ and HSC transplantation, type 2 diabetes treatment, and neurodegenerative diseases. Similar results can be achieve by using cytokines such as IL-7, IL-15, and IL-21 or by activating proper costimulatory receptors like 4-1BB, OX40 and CD27 (21, 204). Combined these studies, it shows that pharmacological, cytokine and costimulatory signal involved stemness can be used to generate antigen-specific stem-like T cells (**Figure 5A**).

Metabolic tuning may be another way to confer stemness to T cells, based on the fact that stem-like T cells own distinct energetic and metabolic characteristics. For example, T cells with stemness show a decrease in mitochondrial membrane potential (ΔΨm) (215, 216). Besides, metabolism is closely related to T cell activity and fate (217).

T_SCM_ cells usually show increased fatty acid oxidation, which further increasing mitochondrial biomass and spare respiratory capacity (218), while show decreased aerobic glycolysis, which devoting to end-effector differentiation. Therefore, strategies based on metabolic taming to directly confer these metabolic signatures to T cell would help harness T_SCM_ cells. Besides, continuous TCR engagement should be control to help maintain long-lived memory CD8^+^ T cells (219). Future study areas of research getting deep insight into the global characterization of the T_SCM_ cell metabolome would lay the better foundation to develop these strategies.

By inducing T_SCM_-specific transcription factors or microRNAs, tumor-specific T_EFF_ cell can be reprogramming to display stem-like features (205, 206) (**Figure 5B**). This direct reprogramming method has also been applied to convert specific mature cells to generate other tissues, such as neurons, heart and liver, or stem-like blood progenitors (220–222).

Ideally, T cell can be de-differentiated into induced pluripotent stem (iPS) cells by inducing expression of stem-related transcription factors including OCT4, SOX2, KLF4 and MYC, then iPS cells can be induced into TN cells by NOTCH activation (207–210) (**Figure 5C**). Therefore, new group of less-differentiated T cells can be re-differentiated from ESC, HSC and iPS cells (211–214). However, the two-step method is now impractical, because its efficiency is limited by low success rate and long duration of reprogramming. Further studies are needed to solve these problems.

T_SCM_ cells have no unique marker. And they shared given markers with T_N_ and memory T cells. This indicates a limitation to isolate pure T_SCM_ cells with high yield for following studies. To address this issue, Lugli et al. developed soring panels containing CD95, CCR7, CD45RA, CD27, CD62L, CD127, CD28 and CD11 (223).

### Investigation methods for cellular contacts promoting stem-like T cells

Given the potential of harnessing T_SCM_ cells, there is much work remained to be done about how cell-cell contact affect the formation of these cells.

Recently, many new technologies have been developed to reveal the relationship between transcriptional factors involved in cellular contact and T cell fate. The most powerful of these contains advances in imaging methods combine with new transcription analyze. One approach to aiming to harness interactions between T cells and DCs is achieved by targeting specific dendritic cell populations (224). This strategies target antigen to restrict presentation to a specific DCs type. The most promising strategy for this purpose is targeting DC-expressed molecules, such as CLEC-9A (expressed on cDC1s) and DEC-205 (expressed on DCs and langerin cells), by antigen to the Fc portion of an antibody heavy chain (224).

By targeting the two molecules with adjuvants, both CD4 positive and CD8 positive T cells show potent cellular responses with strong humoral responses. While without adjuvants, the responses are weak and regulatory T cells are activated (225). Surprisingly, CLEC-9A without adjuvants also show CD4^+^ T responses and humoral responses, which are stronger than DEC-205 targeting without adjuvants (226, 227). The mechanism is unclear about how these targeting strategies induce long-term memory. It is seems that different selection of adjuvants may show different results, which indicates that these strategies are flexible.

NICHE-seq is the most suitable technology to determine cell interactions (228). After dissociation of tissues, cells are separated to identify components of niche, and then investigated by single cell RNA-seq (scRNA-seq). Combined with two-photon laser scanning technology, this method has been used to CD4^+^T cell priming niche in response to viral infection. Further combined with a ligand-receptor bioinformatics analysis, this method widely revealed the cellular interaction within a specific zone (229). This method identifies conjugates of interacting cells before scRNA-seq analysis. Following conjugate RNA-seq, the interacting partners are bioinformatically separated. Applying these approaches to a specific lymphoid niche uncovers what the effects of cell interaction and inflammatory factors to T cell differentiation. Labeling immune cell partnerships by a proximity-dependent labeling system SorTagging intercellular contacts (LIPSTIC), which crosses interfaces of cells, has been developed to investigate CD40-CD40L interactions (230).

In order to study the interaction of ligand and receptors, Staphylococcus aureus transpeptidase sortase A (SrtA) or tag residue are usually used to modified them. When the interact, Based on substrate transfer onto tagged receptor catalyzed by Srt A, the interaction ligand and receptors can be tracked and detected by flow cytometry. Based on this method, someone developed a new method called FucolD, which relying on enzymatic fucosyl-biotinylation, offers alternative choice to label T cells in terms of cell interaction (231). Unlike LIPSTIC, this method is a proximity-based labeling system, allowing detection even without prior known receptor.

Despite that T_SCM_ cells possess superior antitumor response and immune reconstitution capabilities, their rareness and lack of unique markers poses a major hurdle to their clinical application.

A panel was made to include optimal set of markers for human T_SCM_ cells identification (223). Thereby, pure T_SCM_ cells can be identified and separated with high efficiency and yield. It is possible to apply this direct isolation method in clinical application if this process can be completed under GMP grade A condition. However, it requires GMP-grade equipment that integrating with flow sorting function.

**Figure 6:**
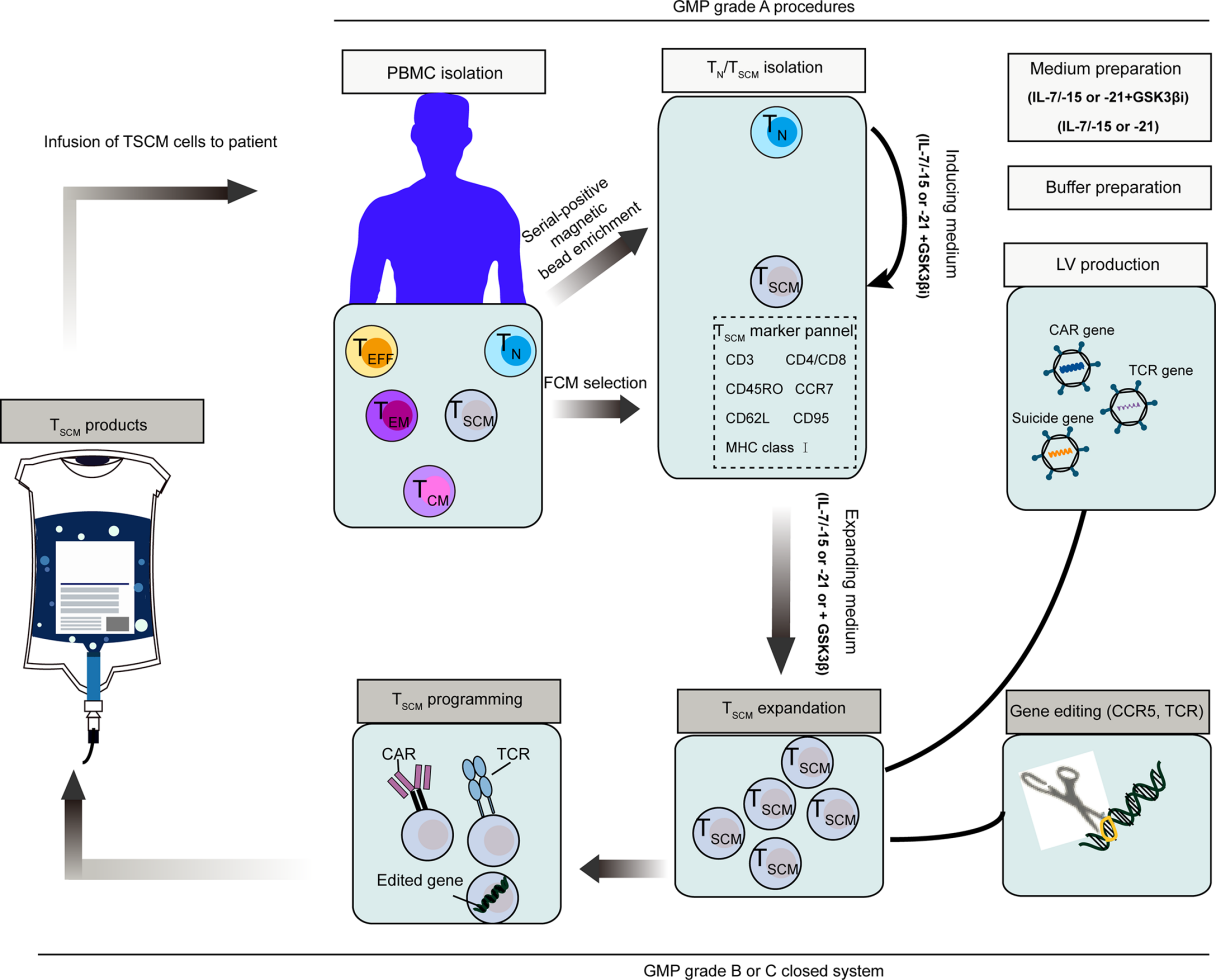
Schematic representation of clinical stem cell memory T (T_SCM_) cells product and programming process (26, 75, 243). Naïve T (T_N)_ cells were then stimulated to transform to T_SCM_ cells with IL-15, IL-21 and IL-7. To maximize the induction of T_SCM_, GSK-3β inhibitor can be used in addition to the above cytokines. T_SCM_ cells can be also isolated directly from PBMCs by a set of markers. Representative programming methods include introducing exogenous genes like specific CAR, TCR and suicide genes, or directly editing endogenous genes. Requirements of GMP grade for main procedures are indicated in the figure. PBMC, peripheral blood mononuclear cells, CAR, chimeric antigen receptor, TCR, T cell receptor; GMP, good manufacturing practice.

It is easier to generate T_SCM_ cells from naive precursors. After selected by magnetic beads, naïve T cells can be induced with IL-15, IL-21 and IL-7, or in combination with small molecular drug (e.g. GS3K GSK-3β inhibitor). These conditions enabled the generation of large number of T_SCM_ cells, which meet clinical needs. A representative scheme that illustrate how to produce and program clinical-grade T_SCM_ cells based on present technologies, is shown in **Figure 6** (26, 75, 223).

## Stem-like T cells associated theraies

### Application in retroviral infections and autoimmune diseases

CD4^+^ T_SCM_ cells might be a new target in patients infected with HIV-1 and HTLV-1. In the context of HIV-1 infection, HIV-1-carried CD4^+^ T_SCM_ cells serve as the source of the virus in HIV-1-infected patients. Although patients received antiretroviral therapy, these CD4^+^ T_SCM_ cells continuously provide progenies infected with HIV-1. There are some essential factors determining self-renewing and expanding abilities of T_SCM_ cells. Therefore, these factors might be new targets to reduce continuous existence of virus in CD4^+^ T_SCM_ cells. For example, targeting WNT-β-catenin pathway, which regulates the homeostasis of T_SCM_ cells (16), might reduce frequency of long-lasting HIV-1-loaed T_SCM_ cells (75) (**Table 2**).

**Table 2:** Overview of researches relating to T_SCM_-cell-based applications for human diseases (21, 21, 26, 75, 232–237).

Disruption		Exploitation	
Methods and techniques	Targeting diseases	Methods and techniques	Targeting diseases
• Wnt antagonists combined with nanoparticle • shRNA targeting TCF7 combined with aptamer technology	• Autoimmunity • T cell leukemia • T cell tropic infections	• Isolation of naïve T cells • *In vitro* T_SCM_ programming • Delivery of T_SCM_ cell to patient • Insertion of specific CAR or TCR • Sensitization by APC loaded with virus-related antigens • Introduction of suicide-genes • Edition of endogenous genes like CCR5	• Cancer • Infectious diseases

Current pharmacological inhibitors for Wnt-β-catenin targeting cancer stem cells can be also applied for eliminate HIV-1 infected CD4^+^ T_SCM_ cells (238). By nanoparticles or aptamer-based targeting systems, it can be more precise to deliver Wnt inhibitors or shRNAs to viral host cells (232, 233) (**Table 2**). Based on similar strategies, T_SCM_ cells can be disturbed in HTLV-1infection followed by T cell leukemia, or in autoimmune diseases.

In addition, ex vivo genetically modifying T_SCM_ cells to make them more resistant to pathogen factors should be another way for the treatment purpose. For instance, knock-out of CCR5, which determines the ability of the virus to enter into host cell (239), therefore imitating the CCR5Δ32 mutation that make hosts resist to HIV-1 (234) (**Table 2**). Once these long-lasting CD4^+^ T cells become intrinsically HIV-1-resistant, they could be utilized to build long-term HIV-resistant immune system. Such population of cells might be helpful to remit HIV-1 infection in absence of drug treatment. Representative clinical trails relating to the adverse effects of T_SCM_ cells are listed in **Supplementary Table 1**.

### Strengthen stemness of T cells for immunotherapy

T_SCM_ cell type is a promising target to promote immunotherapeutic strategies, because it shows several advantages: the robust proliferative potential, the extreme longevity, and the potential to produce various types of the T cells. Adoptive immunotherapy has really become a practical option for cancer treatment (240, 241). Despite some success in patients with advanced cancer, adoptive T cells are not robust enough to induce adequate response, which underscores the need for further improvements (240). Notably, experiments in mice have demonstrated the role of CD8^+^CD45RA^+^CCR7^+^ T_SCM_ cells for supporting long-lasting proliferation of CD19-specific CAR T cells and T cells expressed suicide genes. Similarly, transferring naïve-like CD62L positive T cell population leading to increased number of long-lasting activated T cells, which lead to robust and continuous tumor suppression (16, 109, 242, 243).

T_CM_ cells induce stronger antitumor immunity than highly differentiated T_EM_ cells, and T_SCM_ cells are more profound than T_CM_ cells (244). These robust T_SCM_ cells can be generated *in vitro* and used for clinical exploitation (21, 26) (**Table 2**). Although it is not easy to isolate naive T cells, this step is important to determine the therapeutic effect. Because the differentiation progress of naive T cell would be promoted by co-existed T cells at more differentiated status (245). Relying on the developments in clinical cell-sorting technologies, it is a real strategy to enrich specific cell subsets with high yield under GMP condition (246, 247). The combination strategy of IL-7 and IL-15 is ideal to produce T_SCM_ cells without the need to redirect their specificity. Based on this strategy, naive cell precursors can be programed into tumor-specific, TCR-gene-edited, suicide-gene-modified or virus-specific T_SCM_ cells (21, 235, 248) (**Table 2**).

Additionally, IL-21 also has been used to restrain T cell differentiation and keep their stemness (189), which is realized by activating STAT3 signaling and sustaining the expression of TCF7 and LEF1. A study reported that they induced CAR-modified T_SCM_ cells by use of IL-21 together with inhibitor TWS119, which keep stability of β-catenin resulting in enhanced expression of TCF7 and LEF1 (26). These induced CAR-T cells perform metabolic features of TSCM cells (e.g. low glycolysis and high spare respiratory capacity) (249). CAR-modified T_SCM_ cells can also be attractive for effective treatment of in the setting of chronic virus infection like HIV-1 infection (236, 237) (**Table 2**). Collective studies provide practical approaches for the use of T_SCM_ cells in clinical practice of immunotherapy (250). Based on these approaches, it would be better to confer tumor reactivity to circulating T cells with less-differentiated features, other than directly select specific TILs at exhausted status (251, 252). Overall, the approach that relying on cytokines (such as IL-7, IL-15 and IL-21) is applicable to produce clinical-grade adoptive T_SCM_ cells. And antigen-specific T_SCM_ cells exhibit better clinical outcomes in immunotherapy (**Supplementary Table 1**).

Methods for sorting of specific stem-like T cells also ensure the yield and reproducibility of defined T cell products. Indeed, Unselected PBMC population from individuals with different age, pathogen and therapeutic treatment may show different outcomes owing to varied amplification rate (253–255). Moreover, the benefits of removing more differentiated T cells were offset by simultaneous depletion of T_N_ and T_SCM_ cells (256).

Several studies had successfully converted by infusing T_SCM_ cells derived from circulating CD8^+^ T cells, and it showed robust cellular immune responses in the treatment of different cancers such as non-small cell lung cancer, acute myeloid leukemia and renal cell carcinoma (257–259). T_SCM_ CAR-T cells, which were less likely to become exhausted, exhibited stronger antitumor response in mice model of Raji-ffluc lymphoma (260). Similar observation was achieved in yellow fever-vaccinated patients, when a subset of antigen-specific CD8^+^ T cells was found to display naïve-like phenotypes and play protection effect over 25 years (261). Since T_SCM_ cells are responsible for long-lasting memory of immune effect, T_SCM_ may be an ideal target for prediction and improvement of vaccine effectiveness. Combination use of HPV vaccine with CD40 and TLR3 agonist produces increased number of CD8^+^ T_SCM_ cells, mediating better therapeutic and preventive effects (262).

### Stem-like T cell and immune-checkpoint blockade (ICB)

Despite the potential of ICB to induce long-term remission, most patients fail to achieve durable clinical responses, even in patients with metastatic solid tumors (263–265). Initially, the role of ICBs was thought to rejuvenate exhausted or dysfunctional tumor-infiltrating CD8^+^ T cells. However, to date it is unclear whether dysfunctional tumor-specific TILs can be reversed to activated state by ICB.

Conversely, it has been shown that exhausted CD8^+^ T cells (T_EX_) acquired a steady epigenetic feature different from that of T_EFF_ cells and memory T cells that were minimally remodeled after PD-L1 blockade (266). Preliminary studies have revealed that increased expansion of lymphocytes in blood predicts more potent ICB responses (267), and T cell responses to ICB are derived from newly entered T cells from outside of tumor (268).

In addition, amplification of T cells in blood of patients with cancer can serve as a prediction marker for clinical response against PD-L1 antibody (269). Other studies have shown that tumor-reactive TILs are largely ineffective for ICB-mediated resuscitation prior to ICB. Whether there is a specific subpopulation of T cells determining the ICB responses is still unknown. Other studies identified a subset of TCF1+ CD8+TILs as target T cell group for ICB or vaccination treatment (77, 270). Sade Feldman et al. proved the correlation between TCF1^+^ TILs and enhanced response to ICB (271). But there is no evidence that whether these cells display stable stem-like attributes or not (272).

By scRNA and TCR sequencing, Li’s team studied tumor-reactive TILs in melanoma patients (273). They found that the intratumoral TCF1+ T cells include bystanders without tumor reactivity. Several studies have identified stem-like TCF1+ TILs in human tumors (28, 55, 77, 274). PDL1 blockade could induce CD8+ T cells antitumor effect mediated by TDLNs-derived stem-like PD1+ T cells, which interact with PDL1+ DCs. The existence of PD1-PDL1 interaction in TDLNs other than with tumors predict better outcomes in patients with melanoma (275). However, there may be some tumor-specific TILs that are capable to respond to ICB: those recently entered less-exhausted TCF1+T cells, or T cells that reside in intratumoral niches (50), tertiary lymphoid structures (TLSs) (132, 133), regions of antigen loss or specific metabolic niches (276). Tumeh et al. found that more PD1+CD8+ TILs localized at the margins of invasive tumors, where they interact with PD-L+ cells, are positively correlated with better ICB responses (277). The relationship between the existence of TCF1+ TSL cells in tumors and good prognosis may reflect the properties of these tumors-they have hot TME to promote infiltration of T cells without limitation of tumor antigen specificity. Such tumors were more likely to promote tumor-reactive T cell infiltration from the peripheral tissues, when treated with ICB. Collectively, these studies suggest that the stem-like TCF1+PD1+ T cells in tumor and peripheral compartment may determine ICB responses. They are the source of differentiated T cells in tumor (278). This enlightened researchers to find ways to active and expand stem-like/progenitor exhausted CD8^+^T cells in tumor in order to improve ICB efficacy. Some recent studies have proven to improve anti-PD-1 efficacy by combining with an alarmin HMGN1 peptide or anti-PD-L1 efficacy by combining with cyclophosphamide and vinorelbine (279, 280). These observations above solved an important question about the source of intratumoral stem-like T cellsm that is, they were mainly arised in LNs. Another import question is how they enter the tumor. A recent study pointed out that the existence of tumor-associated endothelial cells (TA-HECs) is essential to recruit stem-like T cells into tumor. And ICB would increase recruitement of stem-like T cells by increasing the maturation of TA-HECs (281). But further studies are still needed to illustrate this question.

On the other hand, ICB still have many shortcomings. Many clinical trials have found that ICB can lead to systemic immune disorders, and long-term treatment can also induce the occurrence of autoimmune diseases (282). In addition, the cessation of PD-1 inhibition may also lead to the enhancement of pathogenic immune responses, which is likely correlated with the loss of immune memory of CD8^+^ T cells (283). Memory CD8^+^ T cells persisting in the body for decades, are antigen-independent, pluripotent, and respond rapidly to secondary aggression. In some chronic viral infections and tumors, effector CD8 T cells alone are not sufficient to induce tumor elimination, and long-lived memory CD8 T cells are required to maintain sustained antitumor immunity. A few studies have found that PD-1 inhibitors reduce memory T cells (284). Therefore, how PD-1 affects memory CD8^+^ T cells deserves further study. Surojit Sarkar’s team found that PD-1 signaling can promote the development and homeostasis of long-lasting memory CD8^+^ T cells by balance metabolic ways relying on glycolysis and fatty acid oxidation. PD-1 deficiency results in memory T cells consumption (285). Although wide type T cells and PD-1 positive T cells had similar numbers of memory precursors during the early activation phase, PD-1-deficient T cells had similar numbers of memory precursors during the contraction and memory maintenance phases of CD8^+^ T cells. Both CD8^+^ T_EFF_ cell numbers were markedly reduced, resulting in an approximately 100-fold reduction in final memory cell numbers in both lymphoid and non-lymphoid regions. The researchers re-stimulated these memory cells with antigen to follow longitudinally *in vivo* secondary responses. After antigen clearance, Loss of PD-1 impairs protection memory of effector T cells and results in the decreased number of antigen-specific cells. At the same time, the researchers also found that PD-1 signaling ensures the normal homeostatic renewal and maintenance of memory CD8 T cells. These results all imply that PD-1 is pivotal in the formation of long-time immune memory of CD8^+^ T cells.

## Conclusions and future directions

Current immunotherapeutic approaches are often inefficient to control the progress of tumors, partly due to negative regulatory factors in TILs and tumor microenvironment. The important role of stem-like T cells has been underlined in recent studies in terms of immunotherapeutic effect of cancer. Therefore, it may take advantages of stem-like T cells to improve antitumor effect, based on complete understanding of how these specific T cells form, maintain and functionalize. We summarized our view about sem-like T cells in **Box 1**.

Additional work highlights the important relationship between several kinds of stem-like T cells and relevant niches, for example, studying interaction between CD8^+^ T cell and stroma, other immune-associated cells and cues provided by lymphatic niches. However, traditional methodology for studying T cell localization like immunohistochemistry limit further investigation due to the fact that the number of antibodies or fluorophores are limited for each imaging. Besides this destructive method disrupt the structure of tissue, losing spatial information of important epitopes. A new technique called CLARITY has been developed to implement antibody labeling and imaging within intact organ (286, 287). Based on multiple staining and visualizing of intact lymph node, it will be easier to elucidate stem-like T cell localization signals and its surrounding microenvironment.

Given the importance of niches to support stem-like T cells, we summarized current knowledge about several specific niches and their spatial location and environmental cues that maintain stemness and promote differentiation. While the secondary lymphoid niches that imprint T_SCM_ cells fate have been illustrated, there is still much work left for further understanding of intratumoral stem-like T cells and niches. This is important and helpful to harness stem-like T cell for therapeutic interventions and develop methods for stem-like T cell generation; either to strength long-lasting T cell responses against chronic infection or cancer, especially for enhancing ICB treatment, or to amplify humoral responses.

## Author contributions

LYi and LY conceived of the topics to include in the review. LYi was mainly responsible to write the review and design the figures. LY provided critical edits to the manuscript. All authors reviewed the manuscript and approved the final version.

## Funding

Funding support was provided by the National Natural Science Foundation of China (No. 81903148, 82073366).

## Conflict of interest

The authors declare that the research was conducted in the absence of any commercial or financial relationships that could be construed as a potential conflict of interest.

## Publisher’s note

All claims expressed in this article are solely those of the authors and do not necessarily represent those of their affiliated organizations, or those of the publisher, the editors and the reviewers. Any product that may be evaluated in this article, or claim that may be made by its manufacturer, is not guaranteed or endorsed by the publisher.
