# Supplementary material for: Stem-like T cells and niches: Implications in human health and disease
Source: Front Immunol. 2022 Aug 17;13:907172. doi: 10.3389/fimmu.2022.907172 (PMC9428355; doi:10.3389/fimmu.2022.907172)
Supplement: Supplementary file 1 [file Table_1.docx]

Supplementary Material

## Supplementary Tables

**Supplementary Table 1**  | Representative stem-like T (TSL) cells involved clinical trials

| No. | Application name | Diseases | Interventions | TSL targeting strategies or therapeutic effects | Location | Phase | ClinicalTrials.gov identifier |
| --- | --- | --- | --- | --- | --- | --- | --- |
| Immunodeficiency diseases | | | | | | | |
| 1 | Safety study of IL-7 in HIV-infected patients | HIV infections  lymphopenia | CYT 107 | IL-7 induced T_SCM_ | Florida,United States etc. | Ⅰ | NCT00477321 |
| 2 | MMF for HIV reservoir reduction | Human immunodeficiency Virus I infection | Mycophenolate Mofetil | MMF reduced T_SCM_ | Washington, United States | II | NCT03262441 |
| 3 | Reducing the residual reservoir of HIV-1 infected cells in patients receiving antiretroviral therapy | HIV infection | Panobinostat  Pegylated Interferon-alpha2a | Antiretroviral therapy reduced T_SCM_ | Massachusetts,  United States | Ⅰ/II | NCT02471430 |
| 4 | Gene therapy for ADA-SCID | Severe combined immunodeficiency syndrome | Gene transduced PBL and/or gene transduced HSC | T_SCM_ contributes to long-lasting gene therapy | Italy | Ⅰ/II | NCT00599781 |
| 5 | ADA gene transfer into hematopoietic stem/progenitor cells for the treatment of ADA-SCID | Immunologic deficiency syndromes | Gene therapy  Busulfan  PEG-ADA | Genetically modified T_SCM_ persists for 12 years | Jerusalem, Israel etc. | II | NCT00598481 |
| 6 | HIV vaccine trial in thai adults | HIV infection | ALVAC-HIV vCP1521^+^ AIDSVAX  ALVAC Placebo ^+^ AIDSVAX Placebo | CD4^+^ T_SCM_ supports innate immunity in  protection against HIV-1 infection | Thailand | Ⅲ | NCT00223080 |
| 7 | Safety and pharmacokinetics (PK) of raltegravir in HIV (human immunodeficiency virus)-infected children and adolescents | HIV infections | Raltegravir | Percentageof CD8^+^ T_SCM_ increased due to combination antiretroviral therapy | California,United States etc. | Ⅰ/II | NCT00485264 |
| Autoimmune disease | | | | | | | |
| 8 | Eltrombopag with standard immunosuppression for severe aplastic anemia | Severe aplastic anemia | Horse ATG ^+^ CsA ^+^ eltrombopag | Identification of CD8^+^ T_SCM_ as biomarker and a therapeutic target in AA | Maryland, United States | Ⅰ/II | NCT01623167 |
| 9 | Sirolimus for autoimmune disease of blood cells | ALPS  Evans syndrome  Idiopathic thrombocytopenic purpura  Autoimmune hemolytic anemia  Autoimmune neutropenia  Systemic lupus erythematosus  Inflammatdisease  Rheumatoid arthritis | Sirolimus (Rapamycin) | Rapamycin is outstanding in the  treatment of autoimmune diseases | Pennsylvania, United States | Ⅰ/II | NCT00392951 |
| 10 | Immune profile in subjects with new onset type 1 diabetes | Diabetes Mellitus, Type 1 | Inguinal lymph node fine needle aspirate biopsy;  Inguinal lymph node core biopsy;  Peripheral blood collection;  Pre- and post-biopsy questionnaire | T_SCM_ exacerbates disease | United Kingdom | Not Applicable | NCT02801942 |
| Tumor | | | | | | | |
| 11 | P-B_CM_A-101 T_SCM_ CAR-T cells in the treatemnt of patients with mutiple myloma | Multiple myeloma | P-B_CM_A-101 CAR-T cells;  Rimiducid | Autologous T stem cell memory (T_SCM_) CAR-T cells | Arizona,United States etc. | Ⅰ/II | NCT03288493 |
| 12 | Administration of anti-CD19-chimeric-antigen-receptor-transduced T cells from the original transplant donor to patients with recurrent or persistent B-cell malignancies after allogeneic stem cell transplantation | Leukemia | Allogeneic stem cell transplant;  Anti-CD19-chimeric-antigen-receptor-transduced T memory stem cells;  Leukapheresis | Inaive precursors activated in the presence of IL-7, IL-21 and the glycogen synthase-3b (GSK-3b) inhibitor TWS119 | Maryland,United States etc. | Ⅰ | NCT01087294 |
| 13 | CD19 CAR-T expressing IL7 and CCL19 combined with PD1 mAb for relapsed or refractory diffuse large B cell lymphoma | Diffuse large B-cell lymphoma | CD19-7×19 CAR-T plus PD1 monoclonal antibody | CD19 CAR-T Expressing IL7 induced T_SCM_ CAR-T | Hangzhou,China etc. | Ⅰ | NCT04381741 |
| 14 | Memory-enriched CAR-T cells Immunotherapy for B Cell lymphoma (MeCAR) | Lymphoma | CD19.CAR-T cells | IL-7/IL-15 induced T_SCM_ CAR-T | Chongqing, China | Ⅰ/II | NCT02652910 |
| 15 | Therapeutic autologous lymphocytes, cyclophosphamide, and Aldesleukin in treating patients With metastatic melanoma | Stage IV Melanoma | Therapeutic autologous lymphocytes;  Aldesleukin;  Cyclophosphamide | IL-21 modulated T_SCM_ | United States, Washington | Ⅰ | NCT01106235 |
| 16 | T cells co- expressing a second generation glypican 3-specific chimeric antigen receptor with cytokines interleukin-21 and 15 as immunotherapy for patients with liver cancer (TEGAR) | Hepatocellular Carcinoma;  Hepatoblastoma | TEGAR T cells;  Cytoxan;  Fludarabine | Interleukin-21 and 15 modulated T_SCM_ | United States | Ⅰ | NCT04093648 |
| 17 | T-Cell infusion, Aldesleukin, and Utomilumab in treating patients with recurrent ovarian cancer | Recurrent ovarian carcinoma | Aldesleukin;  CD8-positive T-lymphocyte;  Leukapheresis;  Utomilumab | 4-1BB agonist induecd T_SCM_ | Texas, United States | Ⅰ | NCT03318900 |
| 18 | Laboratory-treated (central memory/naive) CD8^+^ T cells in treating patients with newly diagnosed or relapsed acute myeloid leukemia | Acute myeloid leukemia | Aldesleukin;  Autologous WT1-TCRc4 Gene-transduced CD8-positive T_CM_/Tn lymphocytes | Naïve CD8^+^ T-derived T_SCM_ | Washington, United States | Ⅰ/II | NCT02770820 |
| 19 | Hotspot TCR-T: a phase I/Ib study of adoptively transferred T-cell receptor gene-engineered T cells (TCR-T) | Malignant epithelial neoplasms | TCR-transduced T cells;  CDX-1140;  Pembrolizumab | CD40 agonist induced T_SCM_ TCR-T | Maryland, United States | Ⅰ | NCT03407040 |
| 20 | T cell receptor immunotherapy targeting NY-ESO-1 for patients with NY-ESO-1 expressing melanoma | Metastatic melanoma | Anti-NY ESO-1 T cell receptor (TCR) cluster of differentiation 62L (CD62L)^+^ cells;  Aldesleukin;  Cyclophosphamide;  Fludarabine | CD62L^+^T-derived T_SCM_ | Maryland, United States | II | NCT02062359 |
| 21 | Immunotherapy of HLA-A2 positive stage III/IV melanoma patients | Melanoma | Vaccination with Melan-A natural and Tyrosinase peptides ^+^ CpG and Montanide adjuvants | T_SCM_ induced by peptide/CpG-B/IFA vaccination | Vaud, Switzerland | Ⅰ | NCT00112229 |
| 22 | C7R-GD2.CAR T cells for patients with GD2-expressing brain tumors | Glioma;  Embryonal Tumor;  Ependymal Tumor | (C7R)-GD2.CART cells; Cyclophosphamide; Fludarabine | Constitutive IL-7 induced T_SCM_ CAR-T | Texas,United States | Ⅰ | NCT04099797 |
| 23 | EBV specific T-lymphocytes for treatment of EBV-positive lymphoma | Lymphoma | Constitutive IL7 (C7R) modified EBV specific T-lymphocytes | Constitutive IL-7 induced T_SCM_ CAR-T | Texas,  United States | Ⅰ | NCT04664179 |
| 24 | CARPALL: immunotherapy with CD19 CAR T-cells for CD19^+^ haematological malignancies | Acute lymphoblastic leukemia;  Burkitt lymphoma | Leukapheresis;  Lymphodepletion with fludarabine  Lymphodepletion with cyclophosphamide  CD19 CAR T-cells | T_SCM_ determines early anti-leukemic responses | United Kingdom | Ⅰ | NCT02443831 |
| 25 | Observational study in patients who underwent an haploidentical transplantation with T-repleted bone marrow | Hematologic malignancy | Post-transplantation cyclophosphamide; Transplantation with T-repleted bone marrow | TN-derived T_SCM_ boosting  immune reconstitution | MI, Italy | Observational Study | NCT02049424 |
| 26 | Reduced intensity conditioning (RIC) regimen and post-transplant cyclophosphamide in haploidentical bone marrow transplantation in in Patients With Poor Prognosis Lymphomas | Lymphoma | Thiotepa;  Fludarabine;  Cyclophosphamide | TN-derived T_SCM_ boosted  immune reconstitution | MI, Italy | II | NCT02049580 |
| 27 | Melanoma vaccine against Neoantigen and shared antigens by CD40 activation and TLR agonists in patients with melanoma | Melanoma | 6MHP;  NeoAg-mBRAF;  PolyICLC;  CDX-1140 | CD40 accelerated the  antigen-specific stem-like Mmemory  CD8^+^ T cells formation | Ohio,United States etc. | Ⅰ/II | NCT04364230 |
